# Supplementary material for: Antiproliferative and Antimigration Activities of Beauvericin Isolated from Isaria sp. on Pancreatic Cancer Cells
Source: Molecules. 2020 Oct 8;25(19):4586. doi: 10.3390/molecules25194586 (PMC7582479; doi:10.3390/molecules25194586)
Supplement: Supplementary file 1 [file molecules-25-04586-s001.pdf]

# Antiproliferative and Antimigration Activities of Beauvericin Isolated from *Isaria* sp. on Pancreatic Cancer Cells

**Hiroaki Yahagi, Tadahiro Yahagi, Megumi Furukawa and Keiichi Matsuzaki ***

School of Pharmacy, Nihon University, 7-7-1 Narashinodai, Funabashi, Chiba 274-8555, Japan; phhi17001@g.nihon-u.ac.jp (H.Y.); yahagi.tadahiro@nihon-u.ac.jp (T.Y.); manyu0907@hotmail.com (M.F.)

* Correspondence: matsuzaki.keiichi@nihon-u.ac.jp; Tel.: +81-47-465-5356

Content

Figure S1. $^{1}H$-NMR spectrum of **1** in $CD_3OD$

........................................................................................S1

Figure S2. $^{13}C$-NMR spectrum of **1** in $CD_3OD$

........................................................................................S2

Figure S3. COSY spectrum of **1** in $CD_3OD$

........................................................................................S3

Figure S4. HMQC spectrum of **1** in $CD_3OD$

........................................................................................S4

Figure S5. HMBC spectrum of **1** in $CD_3OD$

........................................................................................S5

Figure S6. NOE spectra of **1** in $CD_3OD$ (1)

........................................................................................S6

Figure S7. NOESY spectrum of **1** in $CD_3OD$ (2)

........................................................................................S7

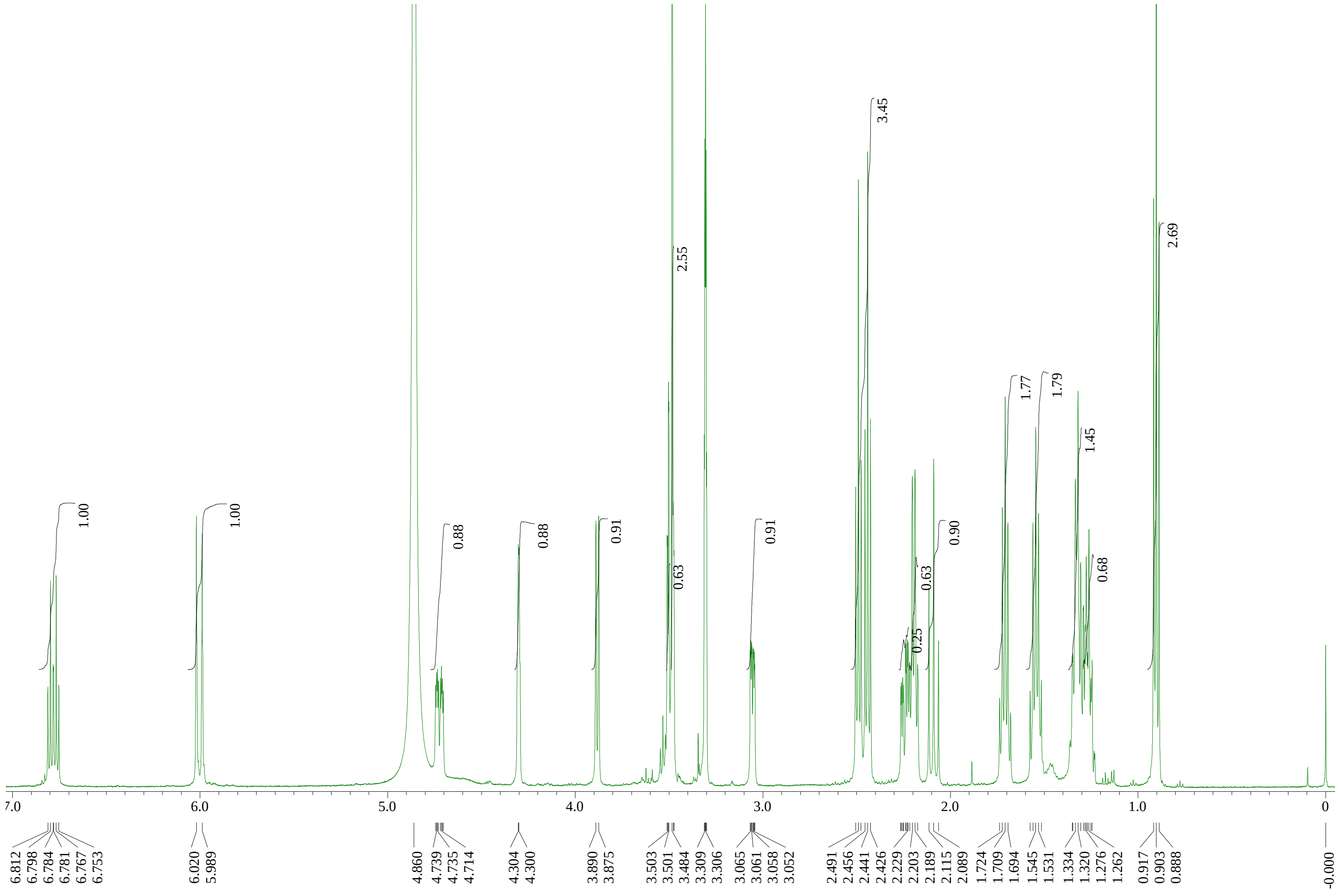

Figure S1. $^{1}H$-NMR spectrum of **1** in $CD_3OD$

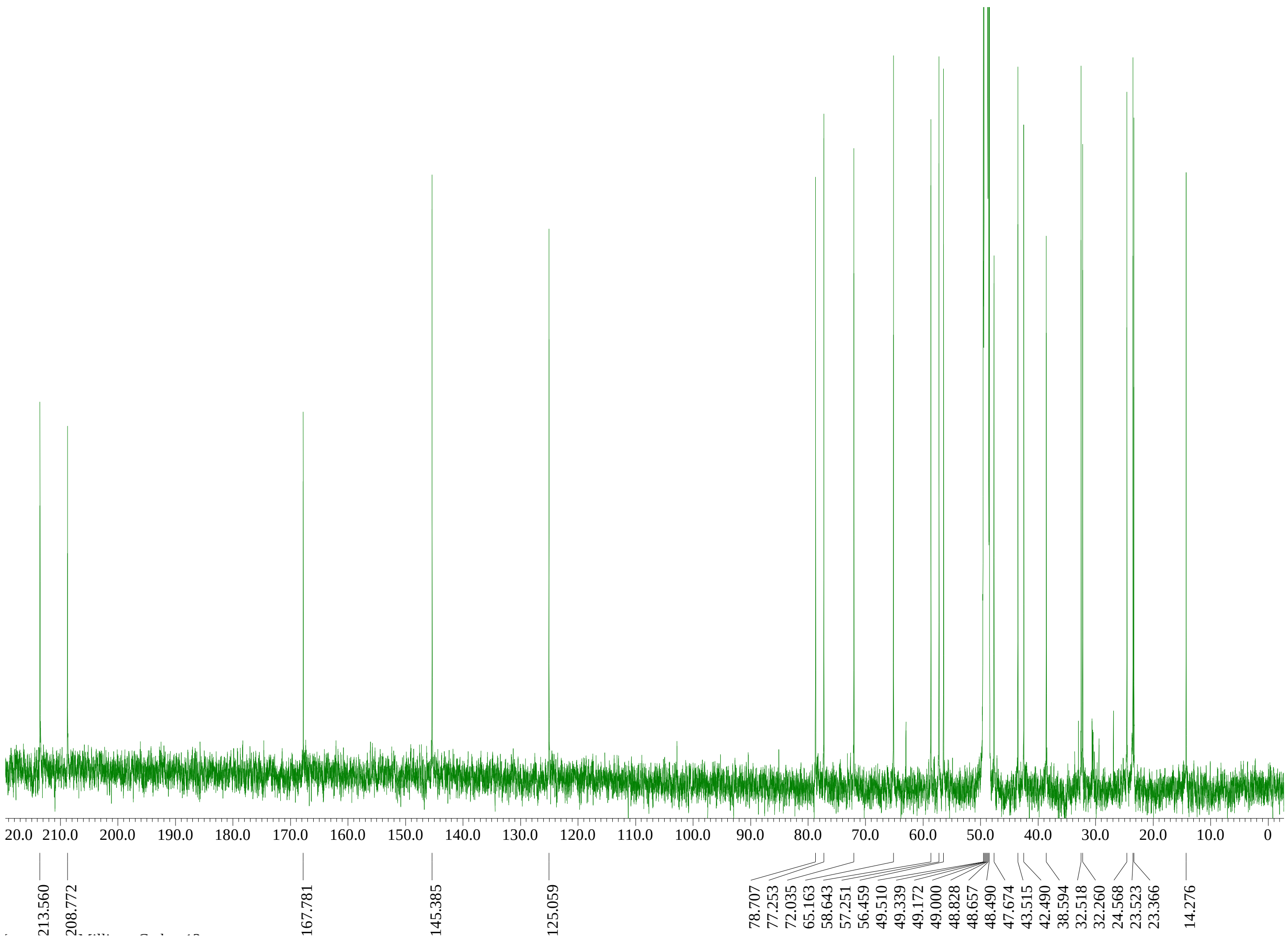


Figure S2. $^{13}C$-NMR spectrum of **1** in $CD_3OD$

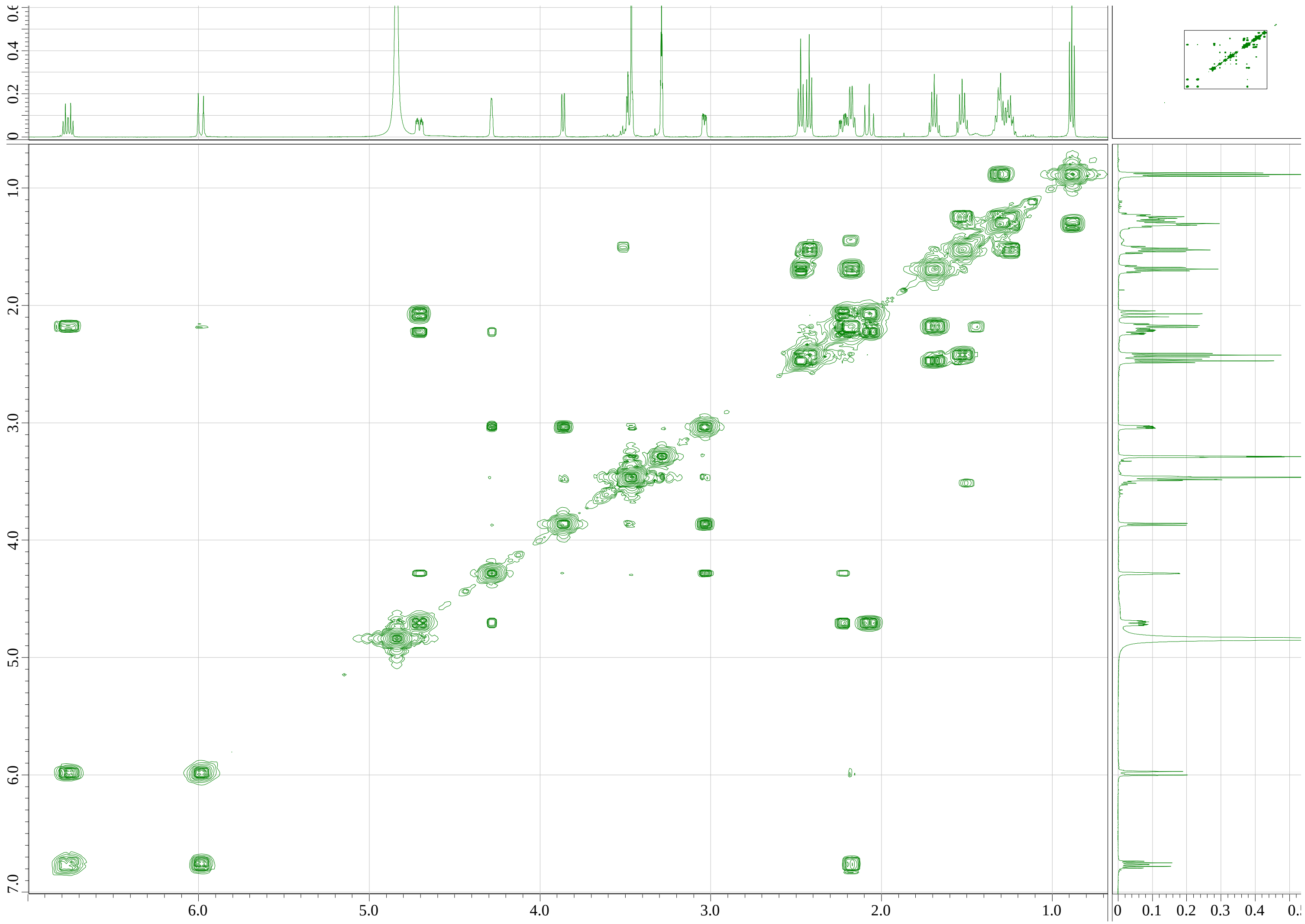


Figure S3. COSY spectrum of **1** in $CD_3OD$

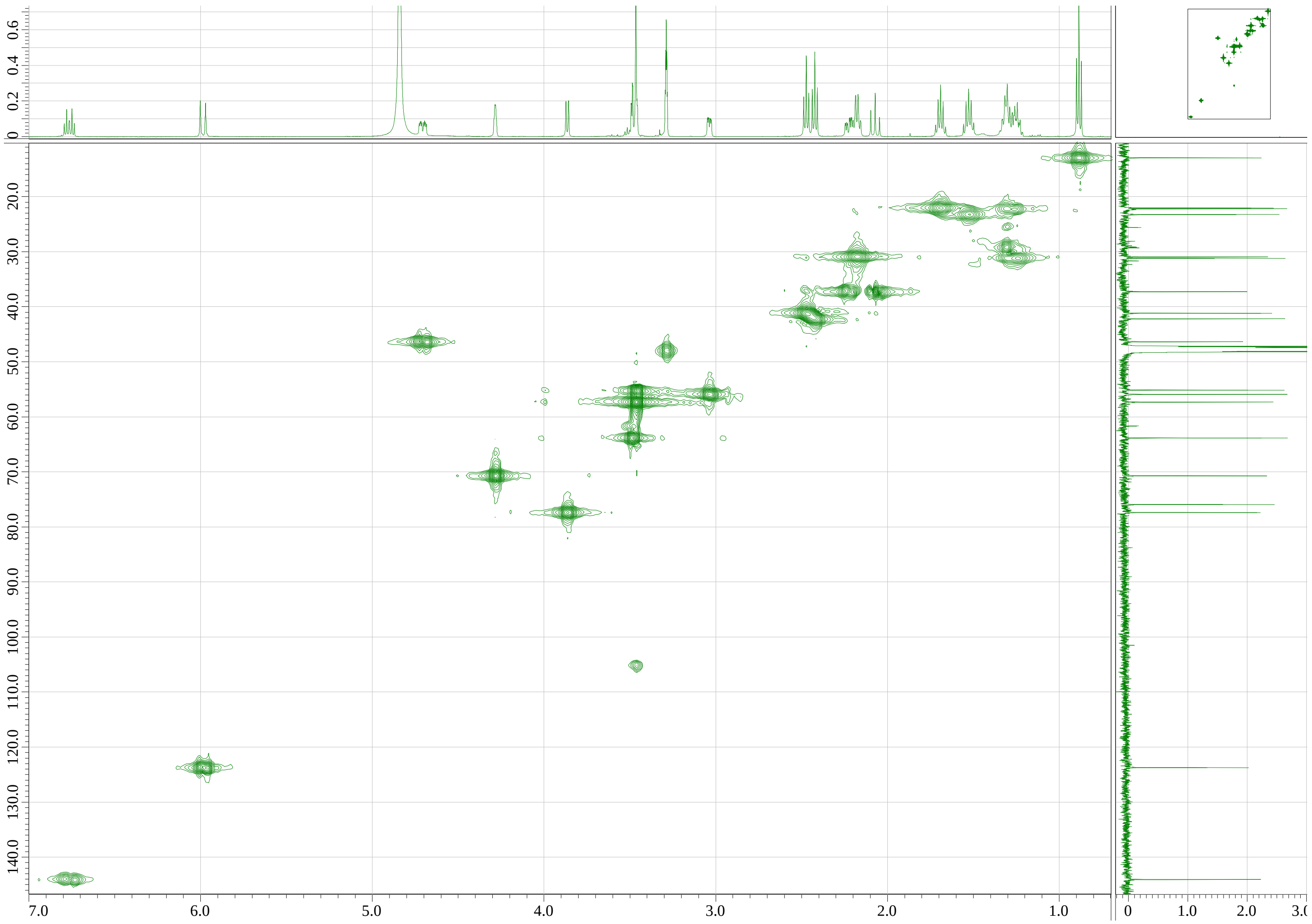


Figure S4. HMQC spectrum of **1** in $CD_3OD$

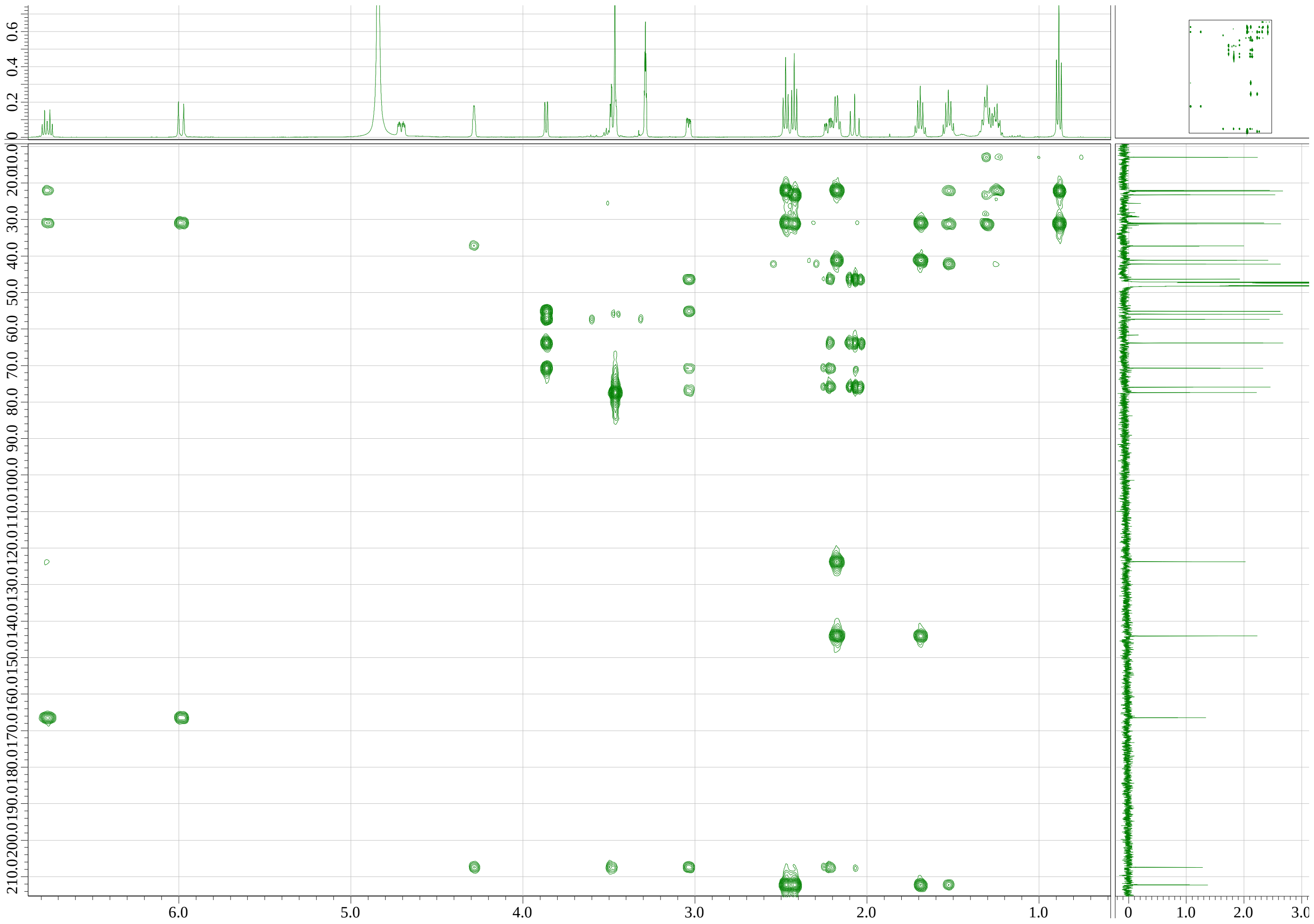


Figure S5. HMBC spectrum of **1** in $CD_3OD$

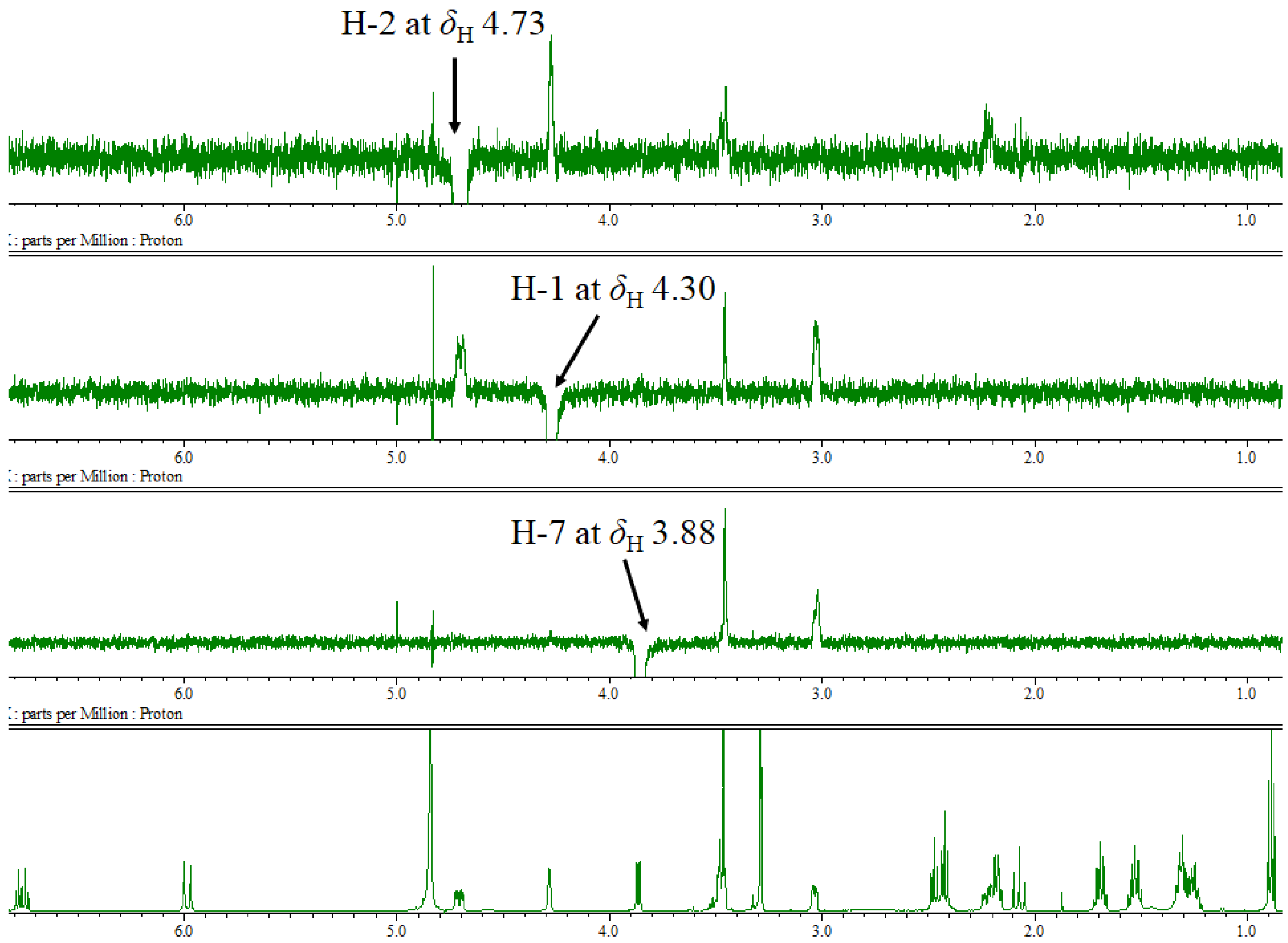


Figure S6. NOE spectra of **1** in $CD_3OD$

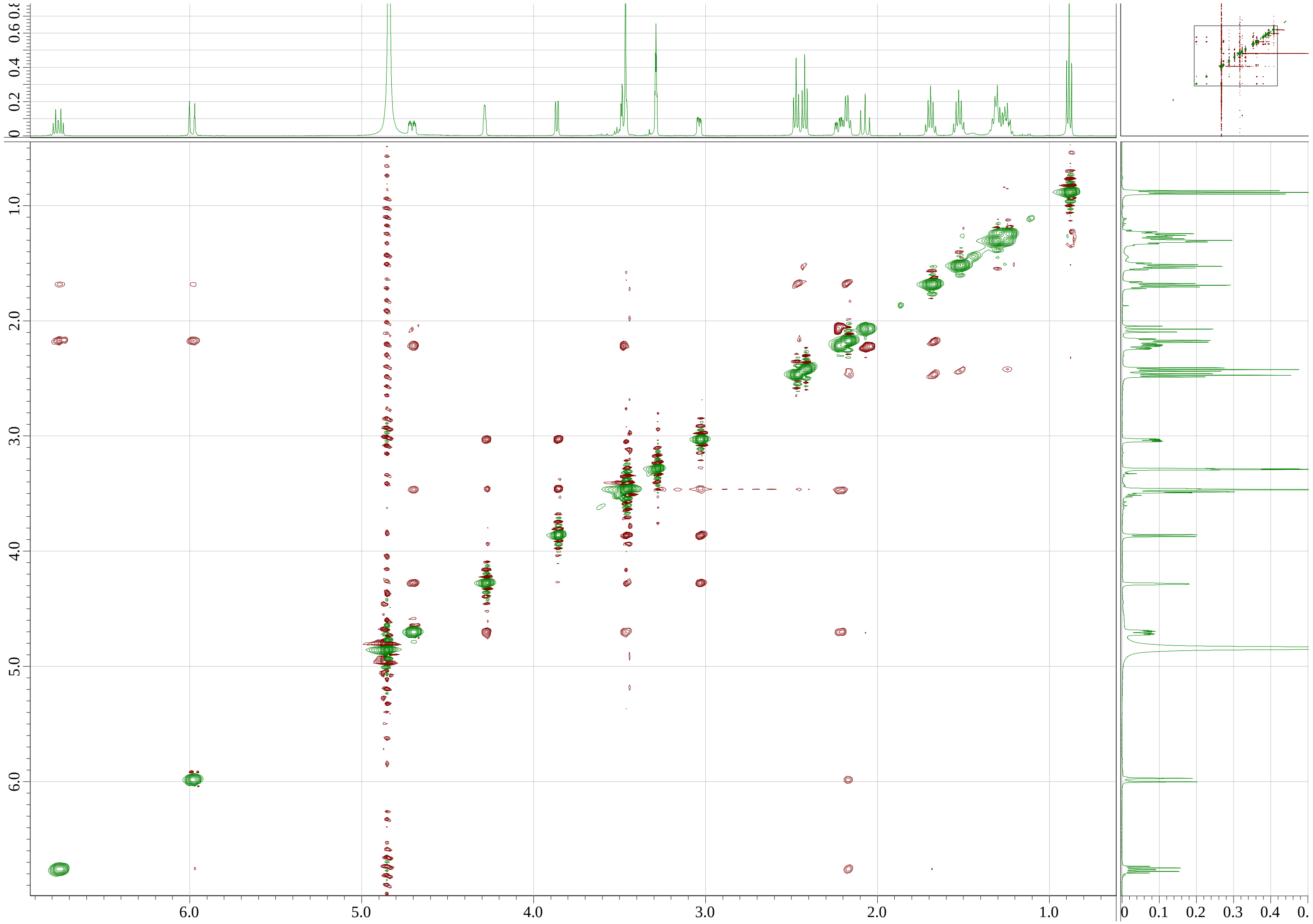


Figure S7. NOESY spectrum of **1** in $CD_3OD$
